# Induced pluripotent stem cell intervention rescues ventricular wall motion disparity, achieving biological cardiac resynchronization post-infarction

**DOI:** 10.1113/jphysiol.2013.252288

**Published:** 2013-04-08

**Authors:** Satsuki Yamada, Timothy J Nelson, Garvan C Kane, Almudena Martinez-Fernandez, Ruben J Crespo-Diaz, Yasuhiro Ikeda, Carmen Perez-Terzic, Andre Terzic

**Affiliations:** 1 Center for Regenerative Medicine, Mayo Clinic Rochester, MN, USA; 2 Marriott Heart Disease Research Program, Division of Cardiovascular Diseases, Departments of Medicine, Molecular Pharmacology and Experimental Therapeutics, and Medical Genetics Mayo Clinic, Rochester, MN, USA; 3 Division of General Internal Medicine, William J. Von Liebig Transplant Center, Mayo Clinic Rochester, MN, USA; 4 Department of Molecular Medicine, Mayo Clinic Rochester, MN, USA; 5 Department of Physical Medicine and Rehabilitation, Mayo Clinic Rochester, MN, USA

## Abstract

Dyssynchronous myocardial motion aggravates cardiac pump function. Cardiac resynchronization using pacing devices is a standard-of-care in the management of heart failure. Post-infarction, however, scar tissue formation impedes the efficacy of device-based therapy. The present study tests a regenerative approach aimed at targeting the origin of abnormal motion to prevent dyssynchronous organ failure. Induced pluripotent stem (iPS) cells harbour a reparative potential, and were here bioengineered from somatic fibroblasts reprogrammed with the stemness factors OCT3/4, SOX2, KLF4, and c-MYC. In a murine infarction model, within 30 min of coronary ligation, iPS cells were delivered to mapped infarcted areas. Focal deformation and dysfunction underlying progressive heart failure was resolved prospectively using speckle-tracking imaging. Tracked at high temporal and spatial resolution, regional iPS cell transplantation restored, within 10 days post-infarction, the contractility of targeted infarcted foci and nullified conduction delay in adjacent non-infarcted regions. Local iPS cell therapy, but not delivery of parental fibroblasts or vehicle, prevented or normalized abnormal strain patterns correcting the decrease in peak strain, disparity of time-to-peak strain, and pathological systolic stretch. Focal benefit of iPS cell intervention translated into improved left ventricular conduction and contractility, reduced scar, and reversal of structural remodelling, protecting from organ decompensation. Thus, in ischaemic cardiomyopathy, targeted iPS cell transplantation synchronized failing ventricles, offering a regenerative strategy to achieve biological resynchronization.

**Key points**

- The pumping function of the heart depends on ordered initiation and propagation of myocardial excitation. Cardiac output is compromised by inconsistent timing and direction of wall motion, leading to dyssynchrony and organ failure.
- Myocardial infarction induces irreversible heart damage. Extensive damage hampers effective pacemaker-based cardiac resynchronization therapy, the current standard-of-care. Establishment of alternative approaches is thus warranted.
- High-resolution imaging was here utilized to non-invasively map suitable therapeutic targets within a dyssynchronous heart. Speckle-tracking echocardiography unmasked the source of progressive cardiac dyssynchrony within the primary infarcted region.
- Bioengineered stem cells with a capacity to induce a regenerative response were implanted into infarcted areas. Speckle-tracking echocardiography and histology assessment revealed that cell therapy achieved cardiac resynchronization and long-term repair.
- This proof-of-concept study thus introduces a stem cell-based regenerative solution to address cardiac dyssynchrony post-infarction.

## Introduction

Cardiac pump function relies on coordinated myocardial motion secured through ordered electromechanical activation (Bers & Harris, 2011). Development of cardiac dyssynchrony accelerates decompensation of heart function, and is commonly associated with progressive organ failure (Kass, 2009). In the setting of myocardial infarction, the discrepancy in myocardial viability between infarcted and non-infarcted areas generates an environment conducive to electrical and mechanical dyssynchrony (Nucifora *et al.* 2010). Florid dyssynchrony has a detrimental impact on ventricular ejection volume, diastolic filling and valve function, precipitating pump failure and leading to poor outcome (Shin *et al.* 2010). Introduction of cardiac resynchronization therapy (CRT) has recently offered a major advance in managing end-stage cardiomyopathic disease. Device-based CRT corrects conduction delays, yet fails to address the origin of contractile deficit (Auricchio & Prinzen, 2011). As a result, the non-viable myocardium remains insufficiently resynchronized by pacing, and dyssynchrony continues uncorrected (Daubert *et al.* 2012). Indeed, a third of patients that received CRT regimens have not responded optimally (Abraham *et al.* 2009; Adelstein *et al.* 2011). Strategies that would afford tissue repair and ensure synchronization of dysfunctional myocardium are thus warranted.

Regenerative interventions are increasingly considered in the management of ischaemic cardiomyopathy (Bartunek *et al.* 2010; Wollert & Drexler, 2010; Penn *et al.* 2011). Multiple candidate cell types have been isolated from cardiac and non-cardiac sources (Janssens, 2010). In this regard, nuclear reprogramming provides an advanced platform to reset cell fate and bioengineer pluripotent stem cells from somatic tissue sources (Yamanaka, 2012). Derived induced pluripotent stem (iPS) cells harbour the potential to form functional cardiac tissue, and to reconstruct heart muscle (Nelson *et al.* 2010; Mauritz *et al.* 2011). To date, however, the impact of iPS cell therapy on cardiac dyssynchrony has not been tested. In the present proof-of-concept study, performed using a murine infarction model, targeted iPS cell transplantation into infarcted myocardial regions restored local wall motion and prevented chronic remodelling achieving cardiac resynchronization.

## Methods

### Ethical considerations

All protocols were carried out under the National Institutes of Health guidelines with approval obtained from the Institutional Animal Care and Use Committee, and the Biosafety Committee at Mayo Clinic. All procedures on living animals were conducted under general inhalation anaesthesia. Animals demonstrating signs of organ failure were removed from the study for humane considerations and sacrificed with carbon dioxide. Following recommendations of the American Veterinary Medical Association and the Institutional Animal Care and Use Committee, all animals were sacrificed with carbon dioxide at the end of the study.

### Bioengineered pluripotent stem cells

**Figure 1:**
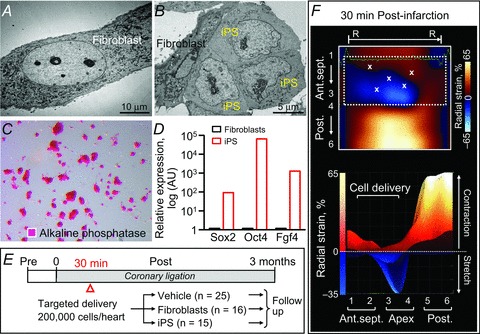
Cell therapy regimen Ultrastructure of fibroblasts (*A*) and derived induced pluripotent stem cells (iPS; *B*) in transmission electron microscopy demonstrating morphological changes from flat fusiform fibroblasts to smaller round iPS with a high nucleus/cytoplasm ratios. Alkaline phosphatase staining (*C*) and gene expression profiling (*D*) confirmed pluripotent reprogramming. *E*, numbers of animals per group are indicated in parentheses. *F*, long-axis radial strain maps deconvoluting infarction-induced regional deformation with superimposed sites of targeted stem cell injection. Upper panel, 2-dimensional view; lower panel, peak strain in 3-dimensional view; X, cell injection sites; R–R, R–R interval; 1, basal anterior-septum (Ant.sept.); 2, mid-Ant.sept.; 3, apical Ant.sept.; 4, apical posterior wall (Post.); 5, mid-Post.; 6, basal Post.

Fibroblasts, i.e. mouse embryonic fibroblasts from a DR-4 strain which is a mixed background of 129/Sv, BALB/c and C57BL/6 strains, served as the somatic tissue source and were transduced with human cDNA encoding the reprogramming factors OCT3/4, SOX2, KLF4 and c-MYC packaged in a lentivirus (Fig. 1*A*–*D*; Nelson *et al.* 2009). Cells were labelled with HIV vectors carrying LacZ (pLenti6/UbC/V5-GW/LacZ, Invitrogen, Grand Island, NY, USA) or luciferase (pSIN-Luc). Pluripotent authenticity and multilineage proficiency were validated in individual iPS cell clones by established *in vitro* (stemness markers expression, metabolic fingerprinting, and embryoid body differentiation), *in vivo* (teratoma formation) and *in utero* (diploid aggregation and contribution to organogenesis) criteria (Martinez-Fernandez *et al.* 2009; Folmes *et al.* 2011). Ultrastructure was examined by transmission electron microscopy (JEOL 1200 EXII, Jeol Ltd, Tokyo, Japan).

### Targeted cell delivery into infarcted regions

**Figure 2:**
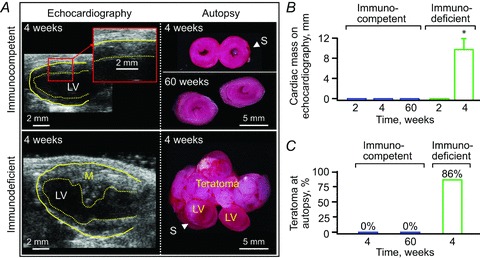
Immunocompetent status defines cell growth outcome Immunocompetent infarcted hearts were free from uncontrolled growth following iPS cell implantation as documented *in vivo* (echocardiography; *A* and *B*) and on autopsy (*A* and *C*) during the 60-week-long follow-up, in contrast to teratoma formation observed in immunodeficient hosts. In *A*: M, mass; LV, left ventricle; S, suture for coronary ligation. In *B*, data represent means ± SEM (*n*= 8 immunocompetent hearts: *n*= 7 immunodeficient hosts); *P < 0.05 *versus* immunocompetent.

Under 1–2% isoflurane anaesthesia, permanent ligation of the left anterior descending coronary artery was performed on male, 8- to 12-week-old C57BL/6 or athymic nude mice (Harlan Laboratories, Indianapolis, IN, USA) as described (Yamada *et al.* 2009; Behfar *et al.* 2010). Pain prophylaxis was implemented by an acetaminophen regimen (100–300 mg kg^−1^ in drinking water) 2 days prior to and 5 days after surgery. Post-ligation, infarcted mice (*n =*56, 17 C57BL/6, 39 athymic nude) were randomized into vehicle-treated (25 athymic nude), fibroblast-treated (*n =*16, 9 C57BL/6, 7 athymic nude), and iPS cell-treated (*n =*15, 8 C57BL/6, 7 athymic nude) cohorts. Fibroblasts or derived iPS cells (200,000 cells per heart in 15 μl propagation media) were delivered into mapped peri-infarcted anterior walls of the left ventricle (LV) by epicardial injection (40,000 cells per site × 5 sites per heart) within 30 min following coronary ligation (Fig. 1*E* and *F*). Cell dose was selected based on previous studies demonstrating that an intracardiac delivery of 200,000 pluripotent stem cells per heart was safe and sufficient to mediate repair (Yamada *et al.* 2008; Nelson *et al.* 2009). Immunocompetent hosts were free from uncontrolled growth up to 60 weeks following iPS cell delivery. In contrast, immunodeficient recipients developed teratoma within 4 weeks as previously reported (Nelson *et al.* 2009), which compromised speckle-tracking and haemodynamics surveillance (Fig. 2). Cohorts demonstrating aneurysmal formation with ejection fraction <25% immediately after infarction were excluded as typical ischaemic cardiac dyssynchrony is characterized by chronic disease progression and dyssynchronous wall motion defined as delayed and reduce peak contraction. Accordingly, functional, structural and electrical endpoints following cell therapy were acquired in an investigator-blinded fashion in immunocompetent hosts. Safety evaluation included daily observation, check of vital signs, electrocardiography, and cell tracking. Systemic histological evaluation upon autopsy was performed at 1 month post-infarction (1 or 2 randomly selected animals in each cohorts), and at the end of the study.

### Ultrasound imaging

Cardiac function and structure were quantified prospectively by echocardiography with a 30 MHz transducer (MS-400; Vevo2100, VisualSonics, Toronto, Canada) up to 3 months post-infarction (Behfar *et al.* 2010). Animals were sedated with 0.5–1.5% isoflurane. Four-limb lead electrocardiograms (Vevo2100 and MP150, Biopac, Goleta, CA, USA) were simultaneously recorded. LV ejection fraction (%) was defined as [(LVVd – LVVs)/LVVd]× 100, where LVVd is LV end-diastolic volume and LVVs; is LV end-systolic volume. LV fractional shortening (%) was calculated as [(LVDd – LVDs)/LVDd]× 100, where LVDd is LV end-diastolic dimension and LVDs is LV end-systolic dimension. The velocity of LV circumferential shortening was derived from the relationship [(LVDd – LVDs)/LVDd]/ET, where ET is ejection time determined by pulse wave Doppler interrogation of the LV outflow tract (Yamada *et al.* 2006).

### Speckle-based deformation mapping and analysis

Regional and global cardiac dynamics, including contractility and synchrony, were deconvoluted by speckle-tracking echocardiography (VisualSonics). Cardiac cycles were acquired digitally from the parasternal long-axis and mid-ventricular short-axis views for assessment of radial, circumferential and longitudinal systolic strain/velocity, and time-to-peak systolic strain/velocity (Bauer *et al.* 2011; Gorcsan & Tanaka, 2011). The LV endocardium was mapped using 48 sampling points that divided the chamber into six segments. In long-axis, the basal anterior-septum, mid-anterior-septum, apical anterior-septum, basal posterior wall, mid-posterior wall and apical posterior segments were defined. In mid-ventricular short-axis, the anterior, anterior-septum, inferior-septum, inferior, posterior, and anterior-lateral segments were further delineated. Validation criteria included stable and continuous endocardial tracking throughout cardiac cycles with a heart rate >380 beats min^−1^ and a frame rate >180 s^−1^ (average heart rate of 478 ± 14 beats min^−1^ and frame rate of 219 ± 11 s^−1^). Ninety-five per cent of sampling points were successfully tracked under pre-established criteria, and further analysed for peak systolic strain/velocity and time-to-peak strain/velocity. Strain (ɛ) was defined as change in length during myocardial contraction and relaxation, and expressed as a percentage: ɛ= (*L*_1_–*L*_0_)/*L*_0_=Δ*L*/*L*_0_, where *L*_0_ is original length, *L*_1_ is final length, and Δ*L* is change in length (Sachdev *et al.* 2011). Tissue contraction patterns were expressed as negative strain values for longitudinal and circumferential motion, and positive values for radial strain. In each segment, peak systolic strain (%) and time-to-peak systolic strain (ms) were analysed. Patterns of abnormal ventricular contractility were classified according to systolic strain magnitude (peak) and timing (initiation and peak of shortening). Specifically, dyssynchrony was defined as a pattern of reduced systolic strain magnitude, early opposite deflection and delayed time-to-peak systole; akinesis as minimal or no contractility with peak systole between −5% and 5%; and dyskinesis as ventricular systolic motion occurring opposite to contraction with peak systolic strain <−5% in radial and >5% in circumferential/longitudinal strain (Carasso *et al.* 2009). Intra-ventricular disparity was quantified by standard deviation of time-to-peak strain/velocity across segments. Stretch was defined as motion occurring in opposite direction to contraction. Systolic total stretch consisted of stretching preceded or not by shortening (De Boeck *et al.* 2009). Stretch-to-shortening ratio (%) was calculated as [(systolic total stretch)/(systolic total shortening)]× 100.

### Cell fate and histology

Cell engraftment and differentiation were tracked by the IVIS 200 Bioluminescence Imaging System (150 mg kg^−1^ D-luciferin i.p., Xenogen, Alameda, CA, USA), and β-galactosidase antibody (1:5000; Abcam, Cambridge, MA, USA) colocalized with α-actinin (1:200; Sigma, St Louis, MO, USA) and 4′,6′-diamidino-2-phenylindole (Molecular Probes) as previously described (Nelson *et al.* 2009). Cell proliferation was evaluated using a Ki67 antibody (1:400; D3B5; Cell Signalling Technology, Danvers, MA, USA). Phosphotungstic acid haematoxylin documentation of scar and Masson's trichrome staining of interstitial fibrosis were quantified by computerized analysis (cellSens 1.3, Olympus, Tokyo, Japan) of 0.5-μm-thick, paraffin-embedded sections (Yamada *et al.* 2009; Behfar *et al.* 2010).

### Statistical analysis

Data are presented as means ± SEM. Paired group analysis was performed using Student's *t* test or non-parametric Mann-Whitney *U* test. Two-way repeated-measures ANOVA was employed for comparison between groups over time (JMP 9, SAS Institute, Cary, NC, USA). Kaplan-Meier analysis with log-rank testing was applied for survival analysis. *P* < 0.05 was predetermined as significant.

## Results

### Targeting ischaemic dyssynchrony by cell therapy

**Figure 3:**
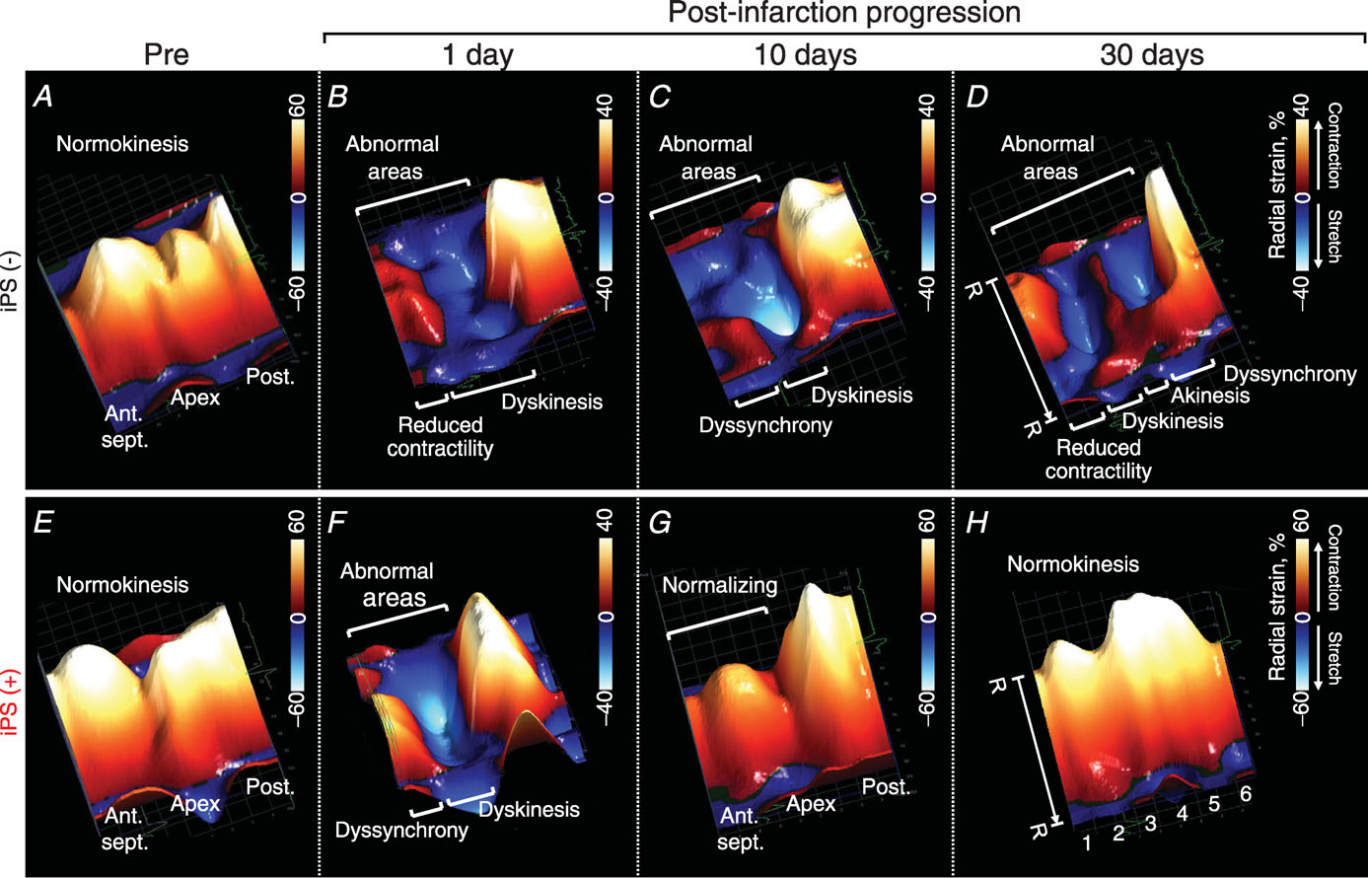
iPS cell implantation normalizes speckle patterns post-infarction In untreated ventricles, long-axis radial strain demonstrated abnormal patterns that progressively extended during follow-up (*A*–*D*). Systolic strain was normalized by iPS cell treatment (*E*–*H*). R–R, R–R interval; 1, basal anterior-septum (Ant.sept.); 2, mid-Ant.sept.; 3, apical Ant.sept.; 4, apical posterior wall (Post.); 5, mid-Post.; 6, basal Post.

**Figure 4:**
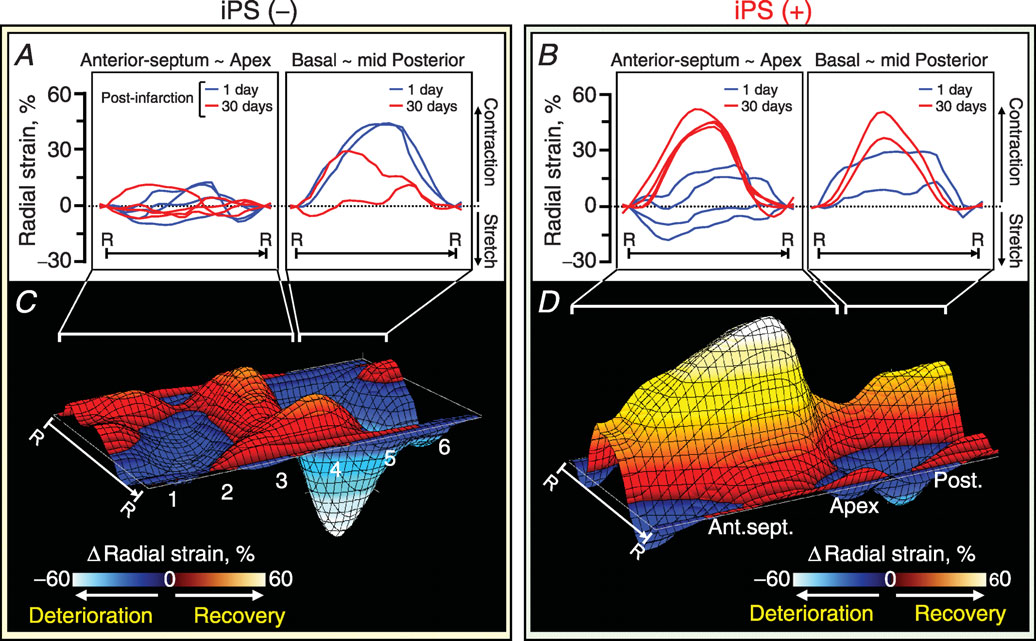
iPS cell-dependent salvage of infarcted and non-infarcted regions Subtraction maps constructed as deviations of long-axis radial strain values from day 1 (blue lines in *A* and *B*) to day 30 post-infarction (red lines in *A* and *B*) in untreated (*C*) and iPS cell-treated (*D*) ventricles. R–R, R–R interval; 1, basal anterior-septum (Ant.sept.); 2, mid-Ant.sept.; 3, apical Ant.sept.; 4, apical posterior wall (Post.); 5, mid-Post.; 6, basal Post.

Advanced echocardiography, applied *in vivo* to murine hearts beating at physiological rates (387–560 beats min^−1^), enabled tracking of tissue speckle patterns (Fig. 3). Speckle tracking is less dependent on the angle of the ultrasound beam and passive wall motion of the heart, complementing more traditional 2-dimensional (2-D) and M-mode echocardiography (Anderson *et al.* 2008). Pre-infarction, systolic radial strain was positive and continuous throughout 48 sampling points of the LV endocardium, indicating homogeneous tissue contraction (Fig. 3*A*). Ligation of the left anterior descending coronary artery precipitated contractile deficit originating from the infarcted anterior-septum and apical segments (day 1; Fig. 3*B*), which progressively advanced into broader deformation and aberrant intra-ventricular disparity (days 10 and 30; Fig. 3*C* and *D*). Despite initial similarity in strain patterns between infarcted hearts with or without cell therapy (Fig. 3*B* and *F*), local delivery of iPS cells into mapped infarcted areas (Fig. 1*F*) gradually salvaged wall motion (Fig. 3*G*), re-introducing normokinesis within 30 days post-infarction (Fig. 3*E*–*H*). Peak systolic strain was >30% in all segments of iPS cell-treated ventricles (Fig. 3*H*), in contrast to the <5% level that persisted in half of the segments within untreated ventricles (Fig. 3*D*). Untreated anterior and apical segments demonstrated loss of contractility with basal and mid-posterior walls manifesting intra-ventricular conduction delay within 30 days post-infarction (Fig. 4*A*). Regional iPS cell intervention restored contractility throughout the mapped segments (Fig. 4*B*). Subtraction maps, constructed as deviations of strain values from days 1 to 30 post-infarction, pinpointed iPS cell-dependent contractile recovery within the infarcted anterior-septum and prevention of malfunction in the non-infarcted posterior wall (Fig. 4*C* and *D*). Thus, the infarcted region is an epicentre of cardiac dyssynchrony and represents a responsive target to iPS cell-based intervention.

### Cell-mediated reversal of initial dysfunction translates into sustained benefit

**Figure 5:**
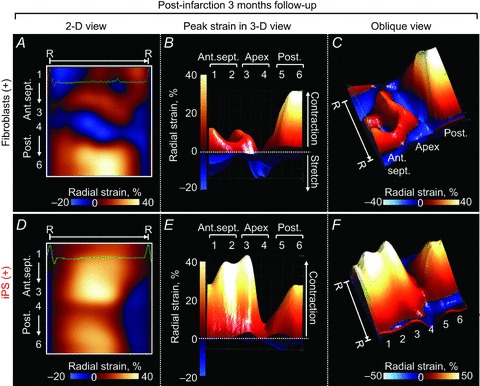
Stable benefit of iPS cell therapy Systolic radial strain, a parameter of tissue contraction was reduced in fibroblast-treated hearts throughout the 3 month follow-up post-infarction (*A*–*C*). In contrast, iPS cell therapy restored contractility during the same observation period (*D*–*F*). R–R, R–R interval; 1, basal anterior-septum (Ant.sept.); 2, mid-Ant.sept.; 3, apical Ant.sept.; 4, apical posterior wall (Post.); 5, mid-Post.; 6, basal Post; 2-D/3-D, 2-/3-dimensional.

**Figure 6:**
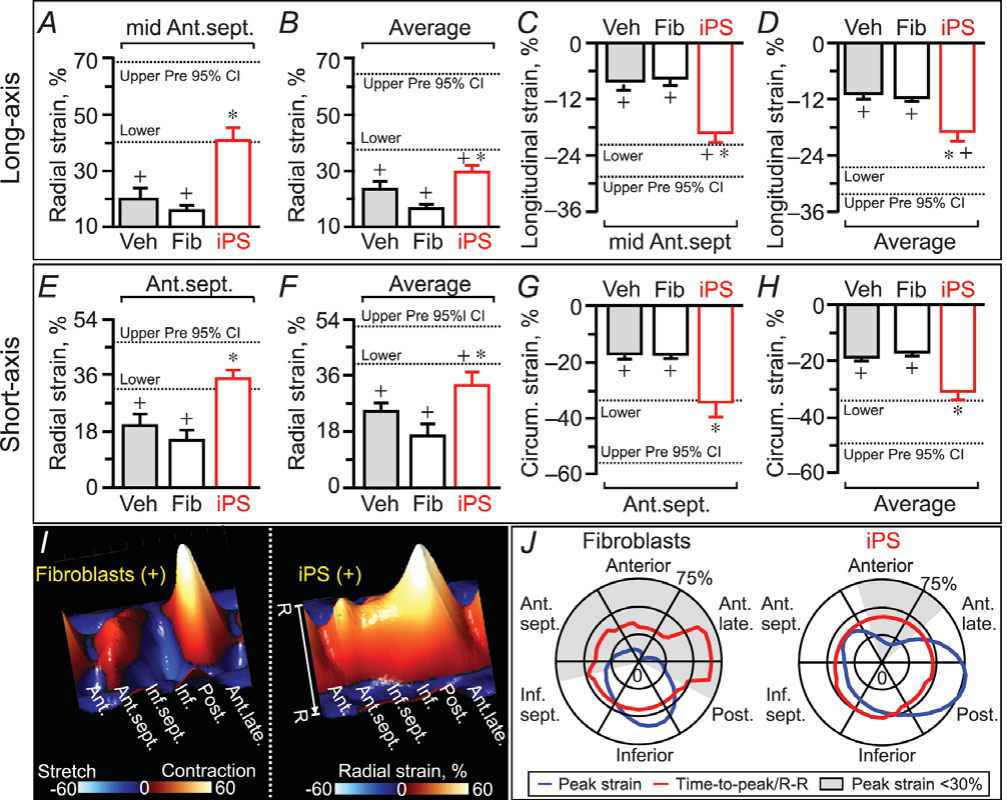
Impact of iPS cell intervention on contractility In *A*–*H*: Ant.sept., anterior-septum; Average, average of 6 ventricular segments; Circum., circumferential; data represent means ± SEM (*n*= 17 in vehicle (Veh), *n*= 6 in fibroblasts (Fib), *n*= 6 in iPS); dotted lines indicate pre-infarction (Pre) 95% confidence interval (CI); ^+^*P* < 0.05 *versus* Pre; *P < 0.05 *versus* Fib. In *I* and *J*: Ant., anterior; Ant.late.,anterior-lateral; Inf., inferior; Inf.sept.,inferior-septum; Post., posterior segments; time-to-peak/R–R, time-to-peak to R–R interval ratio (%).

Over the 3 months follow-up, iPS cell-based intervention produced a stable benefit on tissue contractility across ventricular segments documented in 2-D, 3-D, and oblique views of radial strain (Fig. 5). In contrast, transplantation of fibroblasts demonstrated persistent dysfunction in infarcted zones and paradoxical hypercontractility in non-infarcted areas (Fig. 5*A*–*C*). Within the primary infarcted area, namely the mid-anterior-septum region, peak radial strain from the parasternal long-axis was 16 ± 2% in fibroblast-treated hearts (*n =*6), yet it improved to 41 ± 4% in iPS cell-treated counterparts (*n =*6, *P* < 0.01; Fig. 6*A*). Regional improvement achieved by iPS cell therapy translated into recovery of LV radial strain, which averaged 30 ± 4% across all segments in the iPS cell-treated group (*n*= 6), significantly higher than 17 ± 2% measured in the fibroblast-treated cohort (*n*= 6, *P* < 0.05; Fig. 6*B*). The superior outcome of iPS cell therapy, over fibroblast treatment, was validated by complementary spatial deconvolution. Specifically, longitudinal (Fig. 6*C* and *D*), short-axis radial (Fig. 6*E* and *F*), and circumferential (Fig. 6*G* and *H*) strains all demonstrated significantly improved contractility within infarcted regions leading to functional restitution across the ventricle in iPS cell-treated, but not fibroblast-treated, hearts. Of note, low strain values were equivalent between fibroblast-treated and -untreated cohorts (Fig. 6*A*–*H*), indicating absence of detrimental effects following fibroblast treatment. Thus, iPS cell transplantation rescues myocardial contractility throughout the long-term follow-up period, offering sustained benefit.

### Cell therapy synchronizes wall motions between infarcted and non-infarcted areas

**Figure 7:**
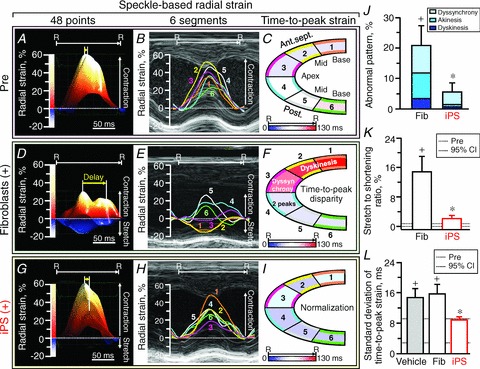
iPS cell-mediated correction of dyssynchrony and discoordination Pre-infarction (Pre), long-axis radial strain was positive in 48-point (*A*) and 6-segment (*B*) mappings with homogeneous contractile timing (*C*). Fibroblast-treated infarcted ventricles (*D*–*F*) were characterized by a decrease in peak strain, and a marked delay between the earliest and the latest contractile timing (yellow arrow), and an exaggerated wall stretch (blue areas in *D*, 1–3 in *E*). In contrast to ineffective fibroblasts (Fib), iPS cell-treated hearts (*G*–*I*) regained similar patterns to Pre, and minimized (*J* and *K*) or normalized (*L*) parameters of mechanical dyssynchrony and/or discoordination. In *A*–*I*: R–R, R–R interval; 1, basal anterior-septum (Ant.sept.); 2, mid-Ant.sept.; 3, apical Ant.sept.; 4, apical posterior wall (Post.); 5, mid-Post.; 6, basal Post. In *J*–*L*: data represent means ± SEM (*n*= 17 in vehicle, *n*= 6 in Fib, *n*= 6 in iPS); dotted lines, pre-infarction (Pre) 95% confidence interval (CI); ^+^*P* < 0.05 *versus* Pre; *P < 0.05 *versus* Fib.

**Figure 8:**
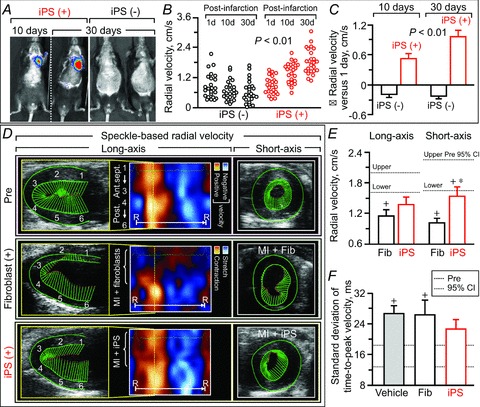
Recovery of homogeneous wall motion in iPS cell-engrafted hearts Engraftment of luciferase-labelled iPS cells (*A*) was consistent with functional recovery of wall motion detected by speckle-based velocity analysis (up to 30 days in *B* and *C*, 3 months in *D*–*F*). In *B*: d, days. In *C*, data represent means ± SEM (*n*= 24 segments in iPS (–), *n*= 24 segments in iPS (+)). In *D*: Fib, fibroblasts; MI, myocardial infarction; R–R, R–R interval; 1, basal anterior-septum (Ant.sept.); 2, mid-Ant.sept.; 3, apical Ant.sept.; 4, apical posterior wall (Post.); 5, mid-Post.; 6, basal Post. In *E* and *F*, numbers of animals *per* group are 17 in vehicle, 6 in Fib, and 6 in iPS; dotted lines, pre-infarction (Pre) 95% confidence interval (CI); ^+^*P* < 0.05 *versus* Pre; *P < 0.05 *versus* Fib.

The difference in myocardial kinesis between infarcted and non-infarcted areas provoked, in the absence of iPS cell therapy, a pronounced disparity of contractile timing (Fig. 6*I*). Time-to-peak strain was markedly prolonged within segments transitioning from the non-contractile anterior-lateral wall to the contractile posterior wall with fibroblast treatment of infarcted hearts, in contrast to an even time-to-peak distribution afforded by iPS cell treatment (Fig. 6*J*). Similarly in long-axis, iPS cell transplantation, but not fibroblast therapy, eliminated the delayed contraction and restored a uniform wall motion across LV segments (Fig. 7). Abnormal strain patterns (aberrant magnitude, timing, and direction of ventricular wall motion), dysfunctional stretch (degree of misdirected contraction), and increase in intra-ventricular delay of time-to-peak strain (heterogeneous contraction timing), which characterized myocardial infarction in the absence of stem cell therapy (Fig. 7*D*–*F*), were prevented or normalized in response to iPS cell treatment (Fig. 7*G*–*J*). Moreover, the stretch-to-shortening ratio significantly decreased (15 ± 4% in fibroblast, *n*= 6; 2 ± 1% in iPS, *n*= 6, *P* < 0.05; Fig. 7*K*), while standard deviation of time-to-peak systolic strain stabilized following iPS cell therapy (16 ± 3 ms in fibroblast, *n*= 6; 9 ± 1 ms in iPS, *n*= 6, *P* < 0.05; Fig. 7*L*). Vector velocity analysis validated the iPS cell-mediated rescue of myocardial contractility and synchrony (Fig. 8). Recovery of contractility matched cell engraftment following iPS cell delivery (Fig. 8*A*–*C*), and was sustained throughout follow-up (Fig. 8*D*–*F*). Thus, by reducing the functional heterogeneity in infarcted and non-infarcted areas, iPS cell therapy prevents mechanical dyssynchrony post-infarction.

### Protection of global cardiac conduction, function and structure

**Figure 9:**
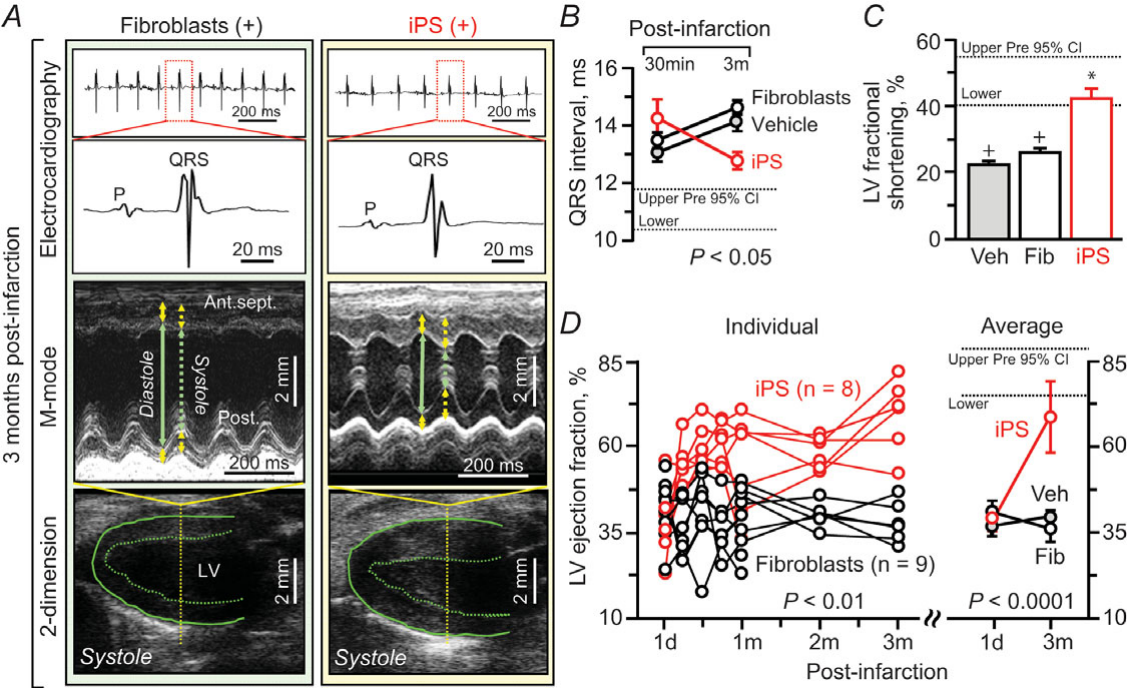
iPS cell therapy improves ventricular conduction and performance post-infarction iPS cell implantation, but not parental fibroblasts (Fib), eliminated cardiac dyssynchrony traits, i.e. wide QRS complex and ventricular dilatation with reduced contractility (*A*). *B*–*D*, benefits of iPS cell treatment over untreated cohorts were significant in this randomized and investigator-blinded study. In *A*: Ant.sept., anterior-septum; LV, left ventricle; Post., posterior wall; green solid/dotted arrow, LV diastolic/systolic dimension; yellow solid/dotted arrow, LV diastolic/systolic wall thickness. In *B*–*D*: d, day; m, months; data represent means ± SEM (*n*= 25 in vehicle (Veh), *n*= 9 in Fib, *n*= 8 in iPS); dotted lines, pre-infarction (Pre) 95% confidence interval (CI); ^+^*P* < 0.05 *versus* Pre; *P < 0.05 *versus* Fib.

**Figure 10:**
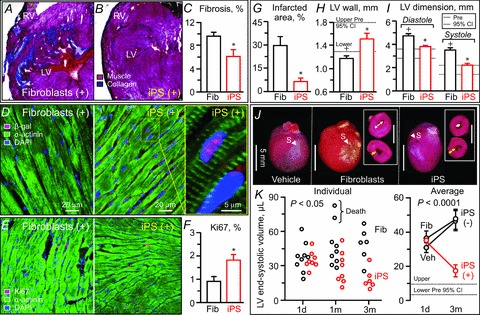
Tissue repair underlies stem cell-based reverse remodelling Histological assessment demonstrated that iPS cells reduced scar formation (*A*–*C*), differentiated and activated cell cycle within the host environment (*D*–*F*), and ultimately averted ventricular dilatation, preventing akinesis and wall thinning post-infarction (*G*–*I*). Stem cell intervention translated into reverse cardiac remodelling *in vivo* (*J* and *K*). Fib, fibroblasts; LV, left ventricle; RV, right ventricle; data represent means ± SEM (*n*= 25 animals in vehicle (Veh), *n*= 9 animals in Fib, *n*= 8 animals in iPS); dotted lines, pre-infarction (Pre) 95% confidence interval (CI); ^+^*P* < 0.05 *versus* Pre; *P < 0.05 *versus* Fib. In *D* and *E*: DAPI, 4′,6′-diamidino-2-phenylindole; β-gal, β-galactosidase. In *J*: white arrow head, suture (S) for coronary ligation; white arrow, LV dimension; yellow arrow, wall thickness of LV free wall; scale bars, 5 mm. In *K*: d, day; m, months.

The benefit of iPS cell treatment on focal infarcted areas impacted the outcome at whole organ level (Fig. 9). QRS prolongation reflecting ventricular conduction delay significantly decreased following iPS cell intervention, contrasting the progressive widening with fragmentation in fibroblast-treated hearts (QRS interval 3 months post-infarction: 14.6 ± 0.6 ms in fibroblast, *n*= 6; 12.8 ± 0.3 ms in iPS, *n*= 6, *P* < 0.01; Fig. 9*A*, top, and *B*). The iPS-treated cohort normalized the LV ejection fraction from 38.9 ± 3.4% (*n*= 8) to 68.7 ± 4.3% at 3 months (*n*= 6, *P <* 0.01; Fig. 9*D*), while fibroblast-treated infarcted ventricles developed systolic dysfunction and chamber dilatation with wall thinning (Fig. 9*A*, left). The LV ejection fraction in the fibroblast-treated cohort maintained abnormal values throughout follow-up (81.9 ± 8.2% at pre-infarction, *n*= 9; 41.0 ± 3.3% at 1 day post-infarction, *n*= 9; 38.2 ± 3.1% at 1 month, *n*= 9; 39.8 ± 1.5% at 2 months, *n*= 6; 36.4 ± 1.6% at 3 months, *n*= 6, *P* < 0.01 *versus* pre-infarction; Fig. 9*D*). Consistent with an improved ejection fraction, fractional shortening (25.7 ± 1.4% in fibroblast, *n*= 6; 42.1 ± 2.8% in iPS, *n*= 6, *P* < 0.01; Fig. 9*C*) and velocity of circumferential shortening (4.7 ± 0.5 circumferences s^−1^ in fibroblast, *n*= 6, 8.2 ± 0.7 circumferences s^−1^ in iPS, *n*= 6, *P* < 0.05) were superior in iPS cell- *versus* fibroblast-treated ventricles. Fibroblast-treated ventricles had extensive scar in the dyssynchronous anterior-septum (Fig. 10*A*). Yet, iPS cell therapy reduced scar burden, which was replaced by remuscularized tissue (fibrosis: 9.6 ± 0.7% in fibroblast, *n*= 30 sections; 6.1 ± 1.2% in iPS, *n*= 15 sections, *P* < 0.05; Fig. 10*B* and *C*). Engraftment of transplanted iPS cells into host ventricles was detected by β-galactosidase expression colocalized with α-actinin (Fig. 10*D*), indicating *in situ* regeneration of the myocardium. Moreover, iPS cell therapy induced cell cycle activation (Fig. 10*E*). The percentage of β-galactosidase-positive or Ki67-positive cells was significantly higher in iPS-treated infarcted hearts, compared with fibroblast-treated cohorts (β-galactosidase: 0.3 ± 0.1% in fibroblast, *n*= 15 sections; 1.4 ± 0.2% in iPS, *n*= 14 sections, *P* < 0.01; Ki67: 0.9 ± 0.2% in fibroblast, *n*= 11 sections, 1.8 ± 0.2% in iPS, *n*= 13 sections, *P*= 0.02; Fig. 10*F*). Tissue repair by iPS cell therapy prevented pathological LV dilatation, wall thinning, and akinesis (Fig. 10*G*–*J*). Collectively, iPS cell-mediated rescue of pump function and structure achieved reversal of LV remodelling (LV end-systolic volume: 1 day post-infarction 36.2 ± 4.3 μl in fibroblast, *n*= 9; 33.9 ± 2.7 μl in iPS, *n*= 8, *P =*0.68; 3 months post-infarction 47.4 ± 5.9 μl in fibroblast, *n*= 6; 17.6 ± 3.6 μl in iPS, *n*= 6, *P* < 0.0001; Fig. 10*K*). The observed benefit was consistent in all individual mice treated with iPS cells (*n*= 8; Figs 9*D* and 10*K*). Cohorts that did not receive cell therapy or received fibroblast treatment showed progressive deterioration (LV end-systolic dimension: 3.34 ± 0.20 mm in infarction without cell therapy; 3.52 ± 0.19 mm with fibroblasts; 2.22 ± 0.17 mm with iPS cells, *P* < 0.01 fibroblasts *versus* iPS cells; Fig. 10*I*–*K*). Two fibroblast-treated animals died prematurely due to systemic heart failure (Fig. 10*K*). The mortality rate at 3 months follow-up was 20% in vehicle-treated (5 deaths out of 25 infarcted animals), 22% in fibroblast-treated (2 out of 9), and 0% in iPS cell-treated (0 out of 8) mice. There was no adverse effect noticed with iPS cell therapy. Thus, targeted iPS cell intervention achieves cardiac resynchronization and reverses remodelling, restoring the global force-generating dynamics in the setting of myocardial infarction.

## Discussion

Therapies that incorporate device-based resynchronization in the management of heart failure are integral to post-infarction care. Indications for device-based resynchronization pacing are chronic, advanced stages of disease diagnosed through global cardiac and systemic evaluation. Yet, practice guidelines do not specify the underlying pathophysiological properties of the failing myocardium amenable to resynchronization (Daubert *et al.* 2012). Despite observed benefit, current practice with pacing regimens indicates a significant portion of non-responders among treated individuals. In particular, scar formation post-infarction is a recognized risk for unfavourable response to device-based cardiac pacing, which critically relies on viable tissue (Mele *et al.* 2009; Riedlbauchová*et al.* 2009; Khan *et al.* 2012). Early interventions that would focus on the origin of disease are thus needed to complement the existing armamentarium for adequate cardiac resynchronization. Accordingly, this study utilized speckle-tracking echocardiography to pinpoint the epicentre of dyssynchrony and used bioengineered pluripotent stem cells for tissue repair. Delivery of iPS cells in the acute phase of infarction selectively eliminated dysfunctional substrates, and achieved long-term resynchronization at the whole organ level. Stem cell-based resynchrony thus emerges as a biotherapeutic strategy to address the primary defects in myocardial pathodynamics underlying cardiac dyssynchrony post-infarction.

### Mechanical dyssynchrony post-infarction resolved *in vivo* by deformation deconvolution

Even following successful reperfusion mechanical dyssynchrony commonly develops within 48 h post-infarction, and is a predictor of adverse ventricular remodelling (Mollema *et al.* 2007; Nucifora *et al.* 2010). Heterogeneous wall motion imposes increased wall stress and triggers neuro-hormonal activation, resulting in pathological remodelling with pump failure and poor outcome (Auricchio & Prinzen, 2008). By fingerprinting tissue speckle patterns, speckle-based strain/velocity analysis offers an *in vivo* non-invasive insight into contractile dynamics (Popović*et al.* 2007). Speckle-based imaging of tissue deformation pinpoints the failing region, and resolves the collapse in cardiac force generation (Bijnens *et al.* 2009). Accordingly, here, high-resolution speckle-tracking echocardiography unmasked the initiation of the disease process. Specifically, the present study identified the transition from the initial focal insult to global dyssynchrony within infarcted murine ventricles. At 3 months, the assessed coronary ligation model displayed dyssynchrony and discoordination of wall motion, QRS widening, low ejection fraction, and chamber dilatation with wall thinning, recapitulating clinical cardiomyopathy traits (Daubert *et al.* 2012). In this way, the murine infarction model used herein combined with advanced imaging provided a relevant experimental foundation to track the onset, progression, and mechanisms of cardiac dyssyncrhony post-infarction.

### iPS cell-based resynchronization underlies benefit post-infarction

Due to a limited capacity for self-renewal, the heart is vulnerable to ischaemic stress and susceptible to organ failure. Discovery of stem cell populations that exhibit the potential to differentiate into specialized tissue types has provided the foundation for cell-based regenerative medicine, boosting innate mechanisms of repair (Bartunek *et al.* 2010; Janssens, 2010). iPS cells harbour a recognized capacity to generate functional cardiac-like beating syncytia expressing contractile proteins and ion channel sets responsive to excitation inputs *in vitro* (Zhang *et al.* 2009; Moretti *et al.* 2010; Nelson *et al.* 2010). Moreover, *in vivo*, iPS cell treatment achieves multilineage tissue reconstruction post-injury (Nelson *et al.* 2009; Mauritz *et al.* 2011; Singla *et al.* 2011). While a number of cell properties may contribute to survival post-transplantation, iPS cells rely on oxygen-independent glycolytic metabolism, providing survival advantage in a low-oxygen environment (Folmes *et al.* 2013). Success of cell therapy for myocardial infarction has been evaluated by cell fate mapping and rescue of heart failure (Wollert & Drexler, 2010). In this study, *in vivo* imaging and histological assessment validated iPS cell engraftment and differentiation, as well as endogenous cell cycle activation within the diseased host heart, and demonstrated reduced fibrosis post-infarction. Moreover, iPS cell treatment restored ejection fraction and prevented ventricular dilatation. Cardiac regional properties are a valuable readout in assessing cell therapies (Menasche, 2011; Petersen *et al.* 2011). Here, iPS cells were locally delivered, within 30 min post-infarction, into hypokinetic or akinetic areas defined by speckle-based strain mapping. Prospective speckle-tracking echocardiography revealed that iPS cell implantation selectively rescued contractility and dyssynchrony in the infarcted regions, leading to global cardiac resynchronization with reverse remodelling. Multiparametric speckle-based analysis demonstrated that recovery of the infarcted anterior-septum preceded rescue of the cardiomyopathic phenotype, suggesting repair of the dyssynchrony substrate as a mechanism for long-term benefit of the iPS cell intervention. While histological assessments identified cell fate within the host environment, *in vivo* wall motion analysis through speckle-tracking echocardiography demonstrated reestablishment of myocardial mechanical properties. In this regard, correction of cardiac wall motion offers an integrated readout of myocardial function achieved by tissue repair through differentiation into cardiomyocytes, vasculature and/or paracrine effects, cell fusion, and the involvement of an innate regenerative response. With no evidence of uncontrolled cell growth or mortality at follow-up in the setting of an allogeneic model, the present study provides initial preclinical demonstration of safe and feasible iPS cell-based resynchronization in the failing infarcted heart. Translation of this proof-of-concept study will require optimization of the stem cell source, methodology of nuclear reprogramming, purging of pluripotent stem cells and securing lineage specific derivatives to eliminate risk of uncontrolled growth, followed by optimization of dose and delivery methods, and tissue implantation. In addition to the differentiation capacity of stem cells, cell–host interaction, immune tolerance and inflammation may all affect cell survival/growth post-transplantation (Nussbaum *et al.* 2007). Potential applications of stem cell-based resynchronization include non-responders to current optimal therapies, and prophylactic early intervention for high-risk groups in heart failure. The concept of biological cell-based resynchronization, as opposed to traditional device-based resynchronization, is supported by recent clinical and preclinical evidence (Chang *et al.* 2008; Herbots *et al.* 2009; van Ramshorst *et al.* 2009; Bonios *et al.* 2011; Pokushalov *et al.* 2011). Due to complexity in the regulation of myocardial mechanics, as well as to the study design focusing on prospective *in vivo* imaging, the relationship of injury, regeneration and resynchronization processes is yet to be fully delineated. Further studies regarding the mechanisms of how to control cardiac wall motion and reconnect cellular and organ physiology will contribute to the establishment of individualized therapeutic protocols in cardiac dyssynchrony post-infarction (Nelson & Terzic, 2011; Gorcsan & Prinzen, 2012; Smith *et al.* 2012).

### Conclusions

Wall motion analysis provides physiological insights into cardiac force generation *in vivo*. Here, high-fidelity speckle-tracking imaging applied to a murine infarction model deconvoluted the evolution of mechanical dyssynchrony and assessed long-term responsiveness to a targeted regenerative intervention. Delivered locally within post-ischaemic dyssynchronous regions, iPS cell therapy synchronized the infarcted tissue and prevented development of refractory ischaemic cardiomyopathy, avoiding organ failure. Biological resynchronization attained through bioengineered stem cell transplantation introduces thereby a novel strategy to preserve cardiac dynamics through tissue repair strategies.

## Translational perspective

Cardiac dyssynchony refers to the disparity of wall motion within the heart, a serious consequence of myocardial infarction associated with poor outcome. Scar formation post-infarction compromises device-based pacing, the current standard-of-care for dyssynchronous heart failure. Stem cells are increasingly considered for cardiac repair. However, the impact of stem cell therapy on cardiac dynamics is largely unknown. This study tested the hypothesis that stem cell transplantation could prevent myocardial damage and restore physiological wall motion, achieving cardiac resynchronization. Bioengineered stem cells (i.e. iPS cells) were delivered into acutely infarcted regions in a murine model. High-resolution speckle-tracking echocardiography unmasked global and regional dynamics of cardiac wall motion *in vivo*, and documented iPS cell-based restoration of synchrony. Compared to progressive dyssynchrony in the absence of stem cell therapy, resychronized hearts post-iPS cell intervention demonstrated improved electrical conduction and pump function, reduced scar, and reversal of structural remodelling. Engraftment and differentiation of implanted iPS cells within the host environment were confirmed by *in vivo* cell tracking and histological evaluation. Adverse effects, including uncontrolled cell growth, were not detected under a titrated cell–dose regimen. Thus, the present study provides initial proof-of-concept suggesting the potential benefit afforded by stem cell-based biological cardiac resynchronization in a model of ischaemic heart failure.

## Abbreviations

Ant.: anterior
Ant.late.: anterior-lateral
Ant.sept.: anterior-septum
β-gal: β-galactosidase
CI: confidence interval
CRT: cardiac resynchronization therapy
DAPI: 4′,6′-diamidino-2-phenylindole
d: days
ET: ejection time
Fib: fibroblasts
Inf.: inferior
Inf.sept.: inferior-septum
iPS: induced pluripotent stem
LV/RV: left/right ventricle
LVDd/LVDs: left ventricular end-diastolic/end-systolic dimension
LVVd/LVVs: left ventricular end-diastolic/end-systolic volume
m: months
MI: myocardial infarction
Post.: posterior wall
R–R: R–R interval
Veh: vehicle
2-D/3-D: 2-dimensional/3-dimensional

## References

[b1] Abraham T, Kass D, Tonti G, Tomassoni GF, Abraham WT, Bax JJ, Marwick TH (2009). Imaging cardiac resynchronization therapy. JACC Cardiovasc Imaging.

[b2] Adelstein EC, Tanaka H, Soman P, Miske G, Haberman SC, Saba SF, Gorcsan J (2011). Impact of scar burden by single-photon emission computed tomography myocardial perfusion imaging on patient outcomes following cardiac resynchronization therapy. Eur Heart J.

[b3] Anderson LJ, Miyazaki C, Sutherland GR, Oh JK (2008). Patient selection and echocardiographic assessment of dyssynchrony in cardiac resynchronization therapy. Circulation.

[b4] Auricchio A, Prinzen FW (2008). Update on the pathophysiological basics of cardiac resynchronization therapy. Europace.

[b5] Auricchio A, Prinzen FW (2011). Non-responders to cardiac resynchronization therapy: the magnitude of the problem and the issues. Circ J.

[b6] Bartunek J, Vanderheyden M, Hill J, Terzic A (2010). Cells as biologics for cardiac repair in ischaemic heart failure. Heart.

[b7] Bauer M, Cheng S, Jain M, Ngoy S, Theodoropoulos C, Trujillo A, Lin FC, Liao R (2011). Echocardiographic speckle-tracking based strain imaging for rapid cardiovascular phenotyping in mice. Circ Res.

[b8] Behfar A, Yamada S, Crespo-Diaz R, Nesbitt JJ, Rowe LA, Perez-Terzic C, Gaussin V, Homsy C, Bartunek J, Terzic A (2010). Guided cardiopoiesis enhances therapeutic benefit of bone marrow human mesenchymal stem cells in chronic myocardial infarction. J Am Coll Cardiol.

[b9] Bers DM, Harris SP (2011). Translational medicine: to the rescue of the failing heart. Nature.

[b10] Bijnens BH, Cikes M, Claus P, Sutherland GR (2009). Velocity and deformation imaging for the assessment of myocardial dysfunction. Eur J Echocardiogr.

[b11] Bonios M, Chang CY, Pinheiro A, Dimaano VL, Higuchi T, Melexopoulou C, Bengel F, Terrovitis J, Abraham TP, Abraham MR (2011). Cardiac resynchronization by cardiosphere-derived stem cell transplantation in an experimental model of myocardial infarction. J Am Soc Echocardiogr.

[b12] Carasso S, Rakowski H, Witte KK, Smith P, Carasso D, Garceau P, Sasson Z, Parker JD (2009). Left ventricular strain patterns in dilated cardiomyopathy predict response to cardiac resynchronization therapy: timing is not everything. J Am Soc Echocardiogr.

[b13] Chang SA, Kim HK, Lee HY, Choi SY, Koo BK, Kim YJ, Sohn DW, Oh BH, Park YB, Choi YS, Kang HJ, Kim HS (2008). Restoration of left ventricular synchronous contraction after acute myocardial infarction by stem cell therapy: new insights into the therapeutic implication of stem cell therapy for acute myocardial infarction. Heart.

[b14] Daubert JC, Saxon L, Adamson PB, Auricchio A, Berger RD, Beshai JF, Breithard O, Brignole M, Cleland J, Delurgio DB, Dickstein K, Exner DV, Gold M, Grimm RA, Hayes DL, Israel C, Leclercq C, Linde C, Lindenfeld J, Merkely B, Mont L, Murgatroyd F, Prinzen F, Saba SF, Shinbane JS, Singh J, Tang AS, Vardas PE, Wilkoff BL, Zamorano JL (2012). EHRA/HRS expert consensus statement on cardiac resynchronization therapy in heart failure: implant and follow-up recommendations and management. Heart Rhythm.

[b15] De Boeck BW, Teske AJ, Meine M, Leenders GE, Cramer MJ, Prinzen FW, Doevendans PA (2009). Septal rebound stretch reflects the functional substrate to cardiac resynchronization therapy and predicts volumetric and neurohormonal response. Eur J Heart Fail.

[b17] Folmes CD, Martinez-Fernandez A, Faustino RS, Yamada S, Perez-Terzic C, Nelson TJ, Terzic A (2013). Nuclear reprogramming with c-Myc potentiates glycolytic capacity of derived induced pluripotent stem cells. J Cardiovasc Transl Res.

[b16] Folmes CD, Nelson TJ, Martinez-Fernandez A, Arrell DK, Lindor JZ, Dzeja PP, Ikeda Y, Perez-Terzic C, Terzic A (2011). Somatic oxidative bioenergetics transitions into pluripotency-dependent glycolysis to facilitate nuclear reprogramming. Cell Metab.

[b18] Gorcsan J, Prinzen FW (2012). Understanding the cardiac substrate and the underlying physiology: Implications for individualized treatment algorithm. Heart Rhythm.

[b19] Gorcsan J, Tanaka H (2011). Echocardiographic assessment of myocardial strain. J Am Coll Cardiol.

[b20] Herbots L, D’hooge J, Eroglu E, Thijs D, Ganame J, Claus P, Dubois C, Theunissen K, Bogaert J, Dens J, Kalantzi M, Dymarkowski S, Bijnens B, Belmans A, Boogaerts M, Sutherland G, Van de Werf F, Rademakers F, Janssens S (2009). Improved regional function after autologous bone marrow-derived stem cell transfer in patients with acute myocardial infarction: a randomized, double-blind strain rate imaging study. Eur Heart J.

[b21] Janssens S (2010). Stem cells in the treatment of heart disease. Annu Rev Med.

[b22] Kass DA (2009). Pathobiology of cardiac dyssynchrony and resynchronization. Heart Rhythm.

[b23] Khan FZ, Virdee MS, Palmer CR, Pugh PJ, O’Halloran D, Elsik M, Read PA, Begley D, Fynn SP, Dutka DP (2012). Targeted left ventricular lead placement to guide cardiac resynchronization therapy: the TARGET study: a randomized, controlled trial. J Am Coll Cardiol.

[b24] Martinez-Fernandez A, Nelson TJ, Yamada S, Reyes S, Alekseev AE, Perez-Terzic C, Ikeda Y, Terzic A (2009). iPS programmed without c-MYC yield proficient cardiogenesis for functional heart chimerism. Circ Res.

[b25] Mauritz C, Martens A, Rojas SV, Schnick T, Rathert C, Schecker N, Menke S, Glage S, Zweigerdt R, Haverich A, Martin U, Kutschka I (2011). Induced pluripotent stem cell (iPSC)-derived Flk-1 progenitor cells engraft, differentiate, and improve heart function in a mouse model of acute myocardial infarction. Eur Heart J.

[b26] Mele D, Agricola E, Galderisi M, Rigo F, Citro R, Dal Monte A, Della Valentina P, Calabrese A, Ferrari R, Study Group of Echocardiography, Italian Society of Cardiology (2009). Echocardiographic myocardial scar burden predicts response to cardiac resynchronization therapy in ischemic heart failure. J Am Soc Echocardiogr.

[b27] Menasche P (2011). Cardiac cell therapy: lessons from clinical trials. J Mol Cell Cardiol.

[b28] Mollema SA, Liem SS, Suffoletto MS, Bleeker GB, van der Hoeven BL, van de Veire NR, Boersma E, Holman ER, van der Wall EE, Schalij MJ, Gorcsan J (2007). Left ventricular dyssynchrony acutely after myocardial infarction predicts left ventricular remodeling. J Am Coll Cardiol.

[b29] Moretti A, Bellin M, Welling A, Jung CB, Lam JT, Bott-Flügel L, Dorn T, Goedel A, Höhnke C, Hofmann F, Seyfarth M, Sinnecker D, Schömig A, Laugwitz KL (2010). Patient-specific induced pluripotent stem-cell models for long-QT syndrome. N Engl J Med.

[b31] Nelson TJ, Martinez-Fernandez A, Terzic A (2010). Induced pluripotent stem cells: developmental biology to regenerative medicine. Nat Rev Cardiol.

[b30] Nelson TJ, Martinez-Fernandez A, Yamada S, Perez-Terzic C, Ikeda Y, Terzic A (2009). Repair of acute myocardial infarction with iPS induced by human stemness factors. Circulation.

[b32] Nelson TJ, Terzic A (2011). Induced pluripotent stem cells: an emerging theranostics platform. Clin Pharmacol Ther.

[b33] Nucifora G, Bertini M, Marsan NA, Delgado V, Scholte AJ, Ng AC, van Werkhoven JM, Siebelink HM, Holman ER, Schalij MJ, van der Wall EE, Bax JJ (2010). Impact of left ventricular dyssynchrony early on left ventricular function after first acute myocardial infarction. Am J Cardiol.

[b34] Nussbaum J, Minami E, Laflamme MA, Virag JA, Ware CB, Masino A, Muskheli V, Pabon L, Reinecke H, Murry CE (2007). Transplantation of undifferentiated murine embryonic stem cells in the heart: teratoma formation and immune response. FASEB J.

[b35] Penn MS, Dong F, Klein S, Mayorga ME (2011). Stem cells for myocardial regeneration. Clin Pharmacol Ther.

[b36] Petersen JW, Forder JR, Thomas JD, Moyé LA, Lawson M, Loghin C, Traverse JH, Baraniuk S, Silva G, Pepine CJ, CCTRN (Cardiovascular Cell Therapy Research Network) (2011). Quantification of myocardial segmental function in acute and chronic ischemic heart disease and implications for cardiovascular cell therapy trials. JACC Cardiovasc Imaging.

[b37] Pokushalov E, Romanov A, Corbucci G, Prohorova D, Chernyavsky A, Larionov P, Terekhov I, Artyomenko S, Kliver E, Shirokova N, Karaskov A, Dib N (2011). Cardiac resynchronization therapy and bone marrow cell transplantation in patients with ischemic heart failure and electromechanical dyssynchrony: a randomized pilot study. J Cardiovasc Transl Res.

[b38] Popović ZB, Benejam C, Bian J, Mal N, Drinko J, Lee K, Forudi F, Reeg R, Greenberg NL, Thomas JD, Penn MS (2007). Speckle-tracking echocardiography correctly identifies segmental left ventricular dysfunction induced by scarring in a rat model of myocardial infarction. Am J Physiol Heart Circ Physiol.

[b39] Riedlbauchová L, Brunken R, Jaber WA, Popová L, Patel D, Lánská V, Civello K, Cummings J, Burkhardt JD, Saliba W, Martin D, Schweikert R, Wilkoff BL, Grimm R, Natale A (2009). The impact of myocardial viability on the clinical outcome of cardiac resynchronization therapy. J Cardiovasc Electrophysiol.

[b40] Sachdev A, Villarraga HR, Frantz RP, McGoon MD, Hsiao JF, Maalouf JF, Ammash NM, McCully RB, Miller FA, Pellikka PA, Oh JK, Kane GC (2011). Right ventricular strain for prediction of survival in patients with pulmonary arterial hypertension. Chest.

[b41] Shin SH, Hung CL, Uno H, Hassanein AH, Verma A, Bourgoun M, Køber L, Ghali JK, Velazquez EJ, Califf RM, Pfeffer MA, Solomon SD, Valsartan in Acute Myocardial Infarction Trial (VALIANT) Investigators (2010). Mechanical dyssynchrony after myocardial infarction in patients with left ventricular dysfunction, heart failure, or both. Circulation.

[b42] Singla DK, Long X, Glass C, Singla RD, Yan B (2011). Induced pluripotent stem (iPS) cells repair and regenerate infarcted myocardium. Mol Pharm.

[b43] Smith NP, McCulloch AD, Paterson DJ (2012). What can modelling provide to cardiac physiology. J Physiol.

[b48] van Ramshorst J, Atsma DE, Beeres SL, Mollema SA, Ajmone Marsan N, Holman ER, van der Wall EE, Schalij MJ, Bax JJ (2009). Effect of intramyocardial bone marrow cell injection on left ventricular dyssynchrony and global strain. Heart.

[b49] Wollert KC, Drexler H (2010). Cell therapy for the treatment of coronary heart disease: a critical appraisal. Nat Rev Cardiol.

[b47] Yamanaka S (2012). Induced pluripotent stem cells: past, present, and future. Cell Stem Cell.

[b44] Yamada S, Kane GC, Behfar A, Liu XK, Dyer RB, Faustino RS, Miki T, Seino S, Terzic A (2006). Protection conferred by myocardial ATP-sensitive K^+^ channels in pressure overload-induced congestive heart failure revealed in *KCNJ11* Kir6.2-null mutant. J Physiol.

[b46] Yamada S, Nelson TJ, Behfar A, Crespo-Diaz RJ, Fraidenraich D, Terzic A (2009). Transplant into pre-implantation embryo yields myocardial infarction-resistant adult phenotype. Stem Cells.

[b45] Yamada S, Nelson TJ, Crespo-Diaz RJ, Perez-Terzic C, Liu XK, Miki T, Seino S, Behfar A, Terzic A (2008). Embryonic stem cell therapy of heart failure in genetic cardiomyopathy. Stem Cells.

[b50] Zhang J, Wilson GF, Soerens AG, Koonce CH, Yu J, Palecek SP, Thomson JA, Kamp TJ (2009). Functional cardiomyocytes derived from human induced pluripotent stem cells. Circ Res.

